# Employment of patients with rheumatoid arthritis - a systematic review and meta-analysis

**DOI:** 10.1186/s41927-023-00365-4

**Published:** 2023-11-14

**Authors:** Lilli Kirkeskov, Katerina Bray

**Affiliations:** 1 Department of Social Medicine, University Hospital Bispebjerg-Frederiksberg, Copenhagen, Denmark; 2 Department of Social Medicine, University Hospital Bispebjerg-Frederiksberg, Nordre Fasanvej 57, Vej 8, Opgang 2.2., 2000 Frederiksberg, Denmark; 3 Department of Occupational and Social Medicine, Holbaek Hospital, Holbaek, Denmark

**Keywords:** Rheumatoid arthritis, RA, Employment rate, Return to work, Unemployment

## Abstract

**Background:**

Patients with rheumatoid arthritis (RA) have difficulties maintaining employment due to the impact of the disease on their work ability. This review aims to investigate the employment rates at different stages of disease and to identify predictors of employment among individuals with RA.

**Methods:**

The study was carried out according to the Preferred Reporting Items for Systematic Reviews and Meta-Analysis (PRISMA) guidelines focusing on studies reporting employment rate in adults with diagnosed RA. The literature review included cross-sectional and cohort studies published in the English language between January 1966 and January 2023 in the PubMed, Embase and Cochrane Library databases. Data encompassing employment rates, study demographics (age, gender, educational level), disease-related parameters (disease activity, disease duration, treatment), occupational factors, and comorbidities were extracted. Quality assessment was performed employing Newcastle–Ottawa Scale. Meta-analysis was conducted to ascertain predictors for employment with odds ratios and confidence intervals, and test for heterogeneity, using chi-square and I^2^-statistics were calculated. This review was registered with PROSPERO (CRD42020189057).

**Results:**

Ninety-one studies, comprising of a total of 101,831 participants, were included in the analyses. The mean age of participants was 51 years and 75.9% were women. Disease duration varied between less than one year to more than 18 years on average. Employment rates were 78.8% (weighted mean, range 45.4–100) at disease onset; 47.0% (range 18.5–100) at study entry, and 40.0% (range 4–88.2) at follow-up. Employment rates showed limited variations across continents and over time. Predictors for sustained employment included younger age, male gender, higher education, low disease activity, shorter disease duration, absence of medical treatment, and the absence of comorbidities.

Notably, only some of the studies in this review met the requirements for high quality studies. Both older and newer studies had methodological deficiencies in the study design, analysis, and results reporting.

**Conclusions:**

The findings in this review highlight the prevalence of low employment rates among patients with RA, which increases with prolonged disease duration and higher disease activity. A comprehensive approach combining clinical and social interventions is imperative, particularly in early stages of the disease, to facilitate sustained employment among this patient cohort.

**Supplementary Information:**

The online version contains supplementary material available at 10.1186/s41927-023-00365-4.

## Background

Rheumatoid arthritis (RA) is a chronic, inflammatory joint disease that can lead to joint destruction. RA particularly attacks peripheral joints and joint tissue, gradually resulting in bone erosion, destruction of cartilage, and, ultimately, loss of joint integrity. The prevalence of RA varies globally, ranging from 0.1- 2.0% of the population worldwide [1, 2]. RA significantly reduces functional capacity, quality of life, and results in an increase in sick leave, unemployment, and early retirement [3–5]. The loss of productivity due to RA is substantial [2, 5–7]. A 2015 American study estimated the cost of over $250 million annually from RA-related absenteeism in United States alone [8].

Research has highlighted the importance of maintaining a connection to the labour market [3, 9], Even a short cessation from work entails a pronounced risk of enduring work exclusion [10]. In Denmark merely 55% on sick leave for 13 weeks succeeded in re-joining the workforce within one year. Among those on sick leave for 26 weeks, only 40% returned to work within the same timeframe [11]. Sustained employment is associated with an improved health-related quality of life [12, 13]. Early and aggressive treatment of RA is crucial for importance in achieving remission and a favourable prognosis reducing the impact of the disease [2, 14–16]. Therefore, initiating treatment in a timely manner and supporting patients with RA in maintaining their jobs with inclusive and flexible workplaces if needed is critical [3, 17].

International studies have indicated, that many patients with RA are not employed [18]. In 2020, the average employment rate across Organization for Economic Co-operation and Development (OECD) countries was 69% in the general population (15 to 64 years of age), exhibiting variations among countries, ranging from 46–47% in South Africa and India to 85% in Iceland [19]. Employment rates were lower for individuals with educational levels below upper secondary level compared to those with upper secondary level or higher education [19]. For individuals suffering with chronic diseases, the employment rates tend to be lower. Prognostic determinants for employment in the context of other chronic diseases encompasses the disease’s severity, employment status prior to getting a chronic disease, and baseline educational level [20–22]. These somatic and social factors may similarly influence employment status of patients with RA. Several factors, including the type of job (especially physically demanding occupations), support from employers and co-workers, social safety net, and disease factors such as duration and severity, could have an impact on whether patients with RA are employed [17, 23, 24]. Over the years, politicians and social welfare systems have tried to improve the employment rates for patients with chronic diseases. In some countries, rehabilitation clinics have been instrumental in supporting patients to remain in paid work. Healthcare professionals who care for patients with RA occupy a pivotal role in preventing work-related disability and support the patients to remain in work. Consequently, knowledge of the factors that contribute to retention of patients with RA at work is imperative [17, 25].

The aim of this study is therefore to conduct a systematic review, with a primary focus on examining employment rates among patients with RA at the onset of the disease, at study entry, and throughout follow-up. Additionally, this study intends to identify predictors of employment. The predefined predictors, informed by the author’s comprehensive understanding of the field and specific to RA, encompass socioeconomic factors such as age, gender, level of education, employment status prior to the disease, disease stage and duration, treatment modalities, and comorbidities, including depression, which are relevant both to RA and other chronic conditions [26].

## Methods

### Protocol

This systematic review was carried out according to Preferred Reporting Items for Systematic Reviews and Meta-Analysis (PRISMA) for studies that included employment rate in patients with rheumatoid arthritis [27]. PROSPERO registration number: CRD42020189057.

### Selection criteria and search strategies

A comprehensive literature search was conducted, covering the period from January 1966 to January 2023 across the PubMed, Embase, and Cochrane Library databases using the following search terms: (Rheumatoid arthritis OR RA) AND (employment OR return to work). Only studies featuring a minimum cohort size of thirty patients and articles in the English language were deemed eligible for inclusion.

The initial screening of articles was based on the titles and abstracts. Studies comprising a working-age population, with current or former employment status, and with no limitations to gender, demographics, or ethnicity were included in this review. Articles addressing topics of employment, work ability or disability, return to work or disability pension were encompassed within the scope of this review. Full-time and part-time employment, but not ‘working as housewives’ was included in this review’s definition of employment. Studies involving other inflammatory diseases than RA were excluded. Reference lists in the selected articles were reviewed, and more articles were included if relevant. A review of the reference lists in the initially selected articles was conducted, with additional articles incorporated if they proved relevant to the research objectives. The eligible study designs encompassed cohort studies, case–control studies, and cross-sectional studies. All other study designs, including reviews, case series/case reports, in vitro studies, qualitative studies, and studies based on health economics were systematically excluded from the review.

### Data extraction, quality assessment and risk-of-bias

The data extraction from the selected articles included author names, year of publication, study design, date for data collection, employment rate, study population, age, gender, educational level, ethnicity, disease duration, and pharmacological treatment. To ensure comprehensive evaluation of study quality and potential bias, quality assessment was independently assessed by two reviewers (LK and KB) using the Newcastle–Ottawa Scale (NOS) for cross-sectional and cohort studies [28]. Any disparities in the assessment were resolved by discussion until consensus was reached. For cross-sectional studies the quality assessment included: 1) Selection (maximum 5 points): representativeness of the sample, sample size, non-respondents, ascertainment of the risk factor; 2) Comparability (maximum 2 points); study controls for the most important, and any additional factor; 3) Outcome (maximum 3 points): assessment of outcome, and statistical testing. For cohort studies the assessment included: 1) Selection (maximum 4 points): representativeness of the exposed cohort, selection of the non-exposed cohort, ascertainment of exposure, demonstration that the outcome of interest was not present at start of study; 2) Comparability (maximum 2 points): comparability of cohorts on the basis of the design or analysis; 3) Outcome (maximum 3 points): assessment of outcome, was the follow-up long enough for outcomes to occur, and adequacy of follow up of cohorts. The rating scale was based on 9–10 items dividing the studies into high (7–9/10), moderate (4–6) or low (0–3) quality. A low NOS score (range 0–3) indicated a high risk of bias, and a high NOS score (range 7–9/10) indicated a lower risk of bias.

### Analytical approach

For outcomes reported in numerical values or percentages, the odds ratio along with their 95% confidence intervals (CI) were calculated, whenever feasible. Weighted means were calculated, and comparisons between these were conducted using t-test for unpaired data. Furthermore, meta-analysis concerning the pre-determined and potentially pivotal predictors for employment status, both at disease onset, study entry, and follow-up was undertaken. The predictors included age, gender, ethnicity, level of education, duration of disease, treatment, and the presence of comorbities, contingent upon the availability of the adequate data. Additionally, attempts have been made to find information regarding on job categorizations, disease activity (quantified through DAS28; disease activity score for number of swollen joints), and quality of life (SF-36 scores ranging from 0 (worst) to 100 (best)). Age was defined as (< = 50/ > 50 years), gender (male/female), educational level college education or more/no college education), race (Caucasian/not Caucasian), job type (non-manual/manual), comorbidities (not present/present), MTX ever (no/yes), biological treatment ever (no/yes), prednisolone ever (no/yes), disease duration, HAQ score (from 0–3)), joint pain (VAS from 1–10), and DAS28 score. Age, disease duration, HAQ score, VAS score, SF36 and DAS28 were in the studies reported by mean values and standard deviations (SD). Challenges were encountered during attempts to find data which could be used for analysing predictors of employment status before disease onset, and at follow-up, as well as factors related to treatments beyond MTX, prednisolone, and biological as predictors for being employed after disease onset. Test for heterogeneity was done using Chi-squared statistics and I^2^, where I^2^ below 40% might not be important; 30–60% may represent moderate heterogeneity; 50–90% substantial heterogeneity; and 75–100% considerable heterogeneity. Meta-analysis for predictors for employment and odds ratio; confidence intervals; and test for heterogeneity were calculated using the software Review Manager (RevMan, version 5.3. Copenhagen: The Nordic Cochrane Centre, The Cochrane Collaboration, 2014).

## Results

### General description of included studies

**Fig. 1:**
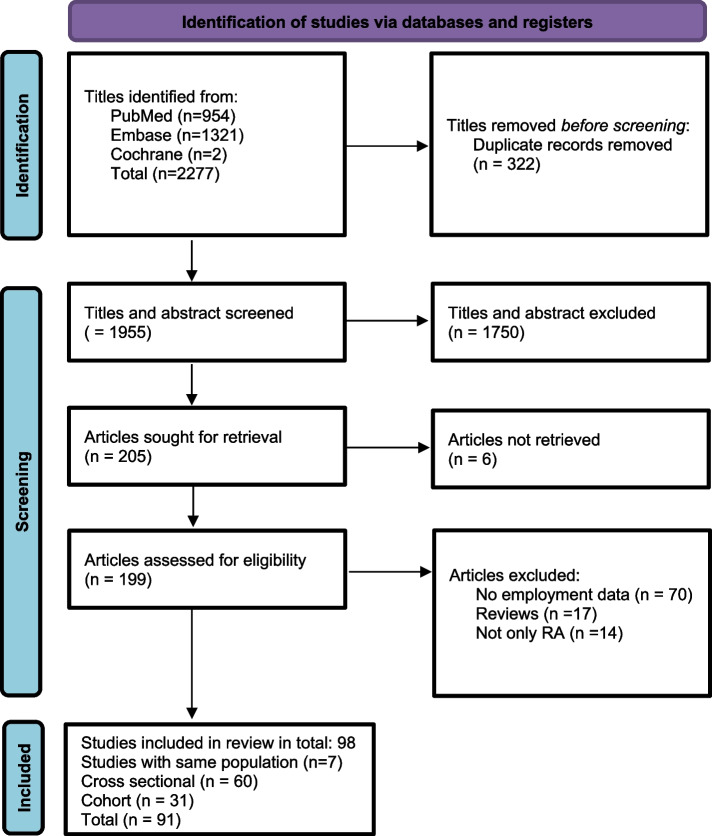
Flow chart illustrating the systematic search for studies examining employment outcome in patients with rheumatoid arthritis

The search yielded a total of 2277 references addressing RA its association with employment. Following the initial title screen, 199 studies were considered relevant for further evaluation. Of those, 91 studies ultimately met the inclusion criteria. Figure 1 shows the results of the systematic search strategy.

Table 1 summarizes the general characteristics of the included studies. The publication year of the included studies ranged from 1971 to 2022. Among the studies, 60 (66%) adopted a cross-sectional research design [13, 18, 29–88, 129] with a total of 41,857 participants analysing data at a specific point in time. Concurrently, 31 studies (34%) adopted a cohort design [89–122, 130] with a total of 59,974 participants. Most of these studies exhibited a small to moderate sample size, with a median of 652 participants. Additionally, single centre studies and studies from high-income countries were predominant. Study details are shown in Table 1.

**Table 1:** Characteristics of the individual studies among patients with rheumatoid arthritis

Reference	Country	Study design	Study population	Disease duration yr, mean	Study period	Participation rate (%)	Age years (mean)	Gender (Female) %	Results Employment rate	Quality assess-ment
Robinson 1971 [108]	Canada	Cohort	*N* = 151 male patients with RA admitted to a Rheumatic disease department, *n* = 94 < 65 yr at follow-up 3.5 yr after discharge	n.a	1958–68	62.3	19–64	0	Baseline: 40% Follow-up: 58%	6
Meenan 1981 [64]	US	Cross sectional	*N* = 245 RA patients from 25 rheumatologists from 19 practices;180 working at disease onset	< 5 yr:42% > 5 yr:58%	n.a	n.a	(52)	67	Disease onset: 74%; 95% male, 65% female Time of study: 30.2%	6
Mäkisara 1982 [63]	Finland	Cross sectional	*N* = 405 RA patients from one hospital	144 5 yr 131 10 yr 130 15 yr	1963–78	n.a	n.a	66.6	5 yr after onset: 60% 10 yr after onset 50% 15 yr after onset 33%	5
Pincus 1984 [103]	US	Cohort	*N* = 75 RA patients followed 9 years	9.8	1973–1982	T1:84.3 T2: 61.8	27–79 (54.7)	71	Age < 55 in 1973 Onset of disease: 75% 1973: 30.6%; 1982:11% Age56-64 in 1973 Onset of disease: 84% 1973: 32%;1982: 4%	5
Kaarela 1987 [98]	Finland	Cohort	*N* = 103 RA patients; 6–9 yr follow-up	7.7	1973–75	60.2	26–64	62	8 yr after onset: Total 44% Full-time 36%; part-time 8%	5
Yelin 1987 [85]	US	Cross sectional	*N* = 306 RA patients from a cohort of 754 RA patients	11	1985	40.6	(51)	72	Yr of diagnosis: 87% 1985: 51%	6
Callahan 1992 [39]	U.S	Cross sectional	*N* = 128 working full-time at disease onset from Vanderbilt and Nashville	10.7	1984–86	n.a	55	41	Full-time 28%; part-time 9.4%	7
Eberhardt 1993, 2007 [92, 93]	Sweden	Cohort	*N* = 84 RA at baseline; *n* = 62 followed 2 years	> 2	n.a	n.a	> 18 (52.4)	65.5	Baseline: 73.8% Follow-up: 53.2%	5
Vlieland 1994 [79]	The Netherlands	Cross sectional	*N* = 138 RA *N* = 127 at follow-up	5–8	1993	92	20–50	100	43.8%	8
Camilleri 1995 [40]	UK	Cross sectional	*N* = 162 RA patients using second line drugs	n.a	n.a	74	< 65	60	30.2%	7
Doeglas 1995 [48]	The Netherlands	Cross sectional	*N* = 292 RA-patients, *n* = 119 working at disease onset, duration of disease < 5 yr	1–2	n.a	79.8	< 65	51.3	Baseline: 40.8% After disease onset:18.5% (45.4% of patients at work at disease onset)	8
Reisine 1995 [130]	US	Cohort	*N* = 497 (T1) employed RA; 392 (T2) five yr follow-up	1- > 10 yr	987-	78.9	(48)	72	Baseline employment: 100% Five yr follow-up: 66%	6
Allaire 1996 [30]	U.S	Cross sectional	*N* = 469 from 44 practices of rheumatologists	7	n.a	47.5	18–64 (47)	78	Full-time 50.4%; part-time 8%	9
Fifield 1996 [50]	US	Cross sectional	*N* = 501 (T1) RA (3-yr follow-up) in work at T1	n.a	n.a	n.a. (T1) 74 (T2)	(47)	70	1 yr of study: 100% 3 yr follow-up: 84%	7
Mau 1996 [100]	Germany	Cohort	*N* = 132 (T1) RA patients; follow-up: *N* = 109 (T2)	7 (T2)	1982–87	82.6	18–60 (49)	73	At follow-up: 47% Highest decline in employment rate during the first 3 yr.’s of disease	6
Van Jaarsveld 1998 [77]	The Netherlands	Cross sectional	*N* = 363 from a cohort of Dutch RA patients	2.8	1990	85.6	19–64	69	RA early patients: 39% General population: 63%	8
Albers 1999 [89]	The Netherlands	Cohort	*N* = 186 early RA patients	3	1991–92	92.1	(53)	61	Female: RA: 23.3% Dutch population: 30.5% Male: RA 51.3% Dutch population: 67.6%	6
De Roos 1999 [46]	US	Cross sectional	*N* = 960 from 15 rheumatologists in 11 cities in 6 states	11.2	n.a	67.8	18–64	77.1	Full-time 36.5%; part-time 9.7%	6
Jäntti 1999 [97]	Finland	Cohort	*N* = 103 RA patients; 83 at 15 yr follow-up; 66 at 20 yr follow-up	8, 15 and 20	1973–1995	n.a	n.a	68	1 yr after RA onset: 69% 15 yr after RA onset: 50% 20 yr after RA onset: 20%	6
Barrett 2000 [90]	UK	Cohort/ cross sectional/ case–control	Cohort 1: *N* = 160 RA; *N* = 110 controls matched for age, sex Cohort 2: *N* = 134	cohort 1: 8.6 cohort 2: 4.1	Employment in 1995 and 1999	45	F:47.8 M:51.6	71.2	Cohort 1: 1995:52.5%; 1999:36.9% Cohort 2: 1999 60.4% 1995: RA:54.4%; controls 74.5%	8
Chorus 2000, 2001, 2003 [13, 42, 43]	Netherlands	Cross sectional	*N* = 1056, a stratified sample of RA patients from a nationwide standardized diagnosis register of rheumatic diseases	11.9	1994–96	62	15–59 (49)	72	At diagnosis 58.3%; at time of study 35.7% (M:56.7%; F:27.7%)	7
Newhall-Perry 2000 [66]	US	Cross sectional	*N* = 150 RA patients from a part of the Western Regional Consortium of Practicing Rheumatologists study form 52 practices	0.5	1993–96	n.a	38–62 (51)	80	52%	7
Young 2000 2002 [115, 116]	UK	Cohort	*N* = 721 RA patients with 5 yr follow-up from rheumatologic clinics in nine districts	0.5	n.a	n.a	> 18	65	Baseline: 48% 5 yr follow-up: 29.3%	6
Reisine 2001 [106]	US	Cohort	*N* = 497 (T1) employed RA; 260 (T2) nine yr follow-up	1—> 10	1988–97	52.3	n.a	70.6	Baseline employment: 100% Nine yr follow-up: 42%	5
Backman 2003 [34]	Canada	Cross sectional	*N* = 239 RA patients from five rheumatologic departments	12.7	n.a	40	18–65 (50)	81	Total 53.1% Full-time:30.5%; part-time 13.0%; 8.4% self-employed	8
Cadena 2003 [38]	Colombia	Cross sectional	*N* = 79 RA from one outpatient clinic	9	2002	n.a	51.5	88.6	Full-time 22.8, part-time 2.5%	4
Kwon 2003 [57]	Korea	Cross sectional	Total population of RA and with-out RA; *N* = 17,311 and a subgroup of 133 RA and 5774 non-RA from the Fourth Korea National Health and Nutrition Survey, KNHANES IV	n.a	2007–9	n.r	> 18 (total) 45–64 (subgroup)		Total: RA 41.7%; non-RA 68.1% Subgroup: RA male: 63.2%; female: 35.3% Non-RA male:83.4%; female 35.2%	7
Lajas 2003 [129]	Spain	Cross sectional	201from a retrospective cohort randomly selected from a rheumatology register in Madrid	7.7	1997	58.8	(64.3)	78	62.1%	6
Vlak 2003 [113]	Israel	Cohort	*N* = 188 RA (95 receiving and 93 not receiving disease modifying antirheumatic drugs, DMARD), RCT with 42 months follow-up	5–8	n.a	n.a	58/50	91/81	DMARD: Baseline: 20.5%; follow-up:8.6% Non-DMARD: Baseline:44.3%; follow-up:22.1%	6
Yelin 2003 [114]	US	Cohort	*N* = 497 Two cohorts of RA patients (1999); 238 receiving; 259 not receiving etanercept	> = 3	1999	Cohort 1: 58.9 Cohort 2: 46.7	18–64	84	Receiving/not receiving etanercept: At time of diagnosis: 75%/77% Follow-up: 56%/63%	6
Dadoniene 2004 [45]	The Netherlands	Cross sectional	*N* = 238 RA patients from a RA-register in Vilnius	10.4	1998-	58	16–65 (52.2)	86	37%	7
LaCaille 2004 [58]	Canada	Cross sectional, retrospective	581 RA patients using a province-wide treatment program	n.a	1991–98	52	18–65 (47–48)	78–83	Onset of RA 65% 1 yr after onset: 57.5% 2 yr after onset: 55.6% 5 yr after onset: 47% 10 yr after onset: 38%	7
Poulakka 2004, 2005 [104, 105]	Finland	Cohort	*N* = 162 RA patients randomly assigned to receive either a combination therapy (*n* = 82) or single therapy (*n* = 80) (with or without prednisolone)-five yr follow-up	0.5	1993–95 + 5 yr follow-up	83.1	(45)	62.3	Baseline employment: Single-drug therapy: 82% Combination therapy: 89% 5 yr follow-up: Single-drug therapy: 52.4% Combination therapy: 68.8%	6
Allaire 2008 [31]	U.S	Cohort longitudinal	*N* = 5384 from National Data Bank longitudinal study of RA, diagnosed by rheumatologists	n.a	2002- 5	88	18–64 (51.3)	81.9	Disease onset: 84.6% Currently: Total 58,5% Full-time 48.3%	8
Verstappen 2005 [78]	The Netherlands	Cross sectional	*N* = 296 from 7 outpatient clinics	4.3	1990–98	82	25–65	73	RA patients/general population Total: 43%; 72.2% Males: 58.8%; 83.4% Female: 37.1%/60.7%	8
Chung 2006 [44]	Finland US	Cross sectional	*N* = 269 RA-patients from US and *N* = 364 from Finland, in employment at disease onset	3–4	2001–2	n.a	< 65 (46–47)	U.S:72.5 Finl:70.9	At disease onset: US:88.5%; Fi:65.4% At time of study: US: 1,2,3,4 yr:81.4,78.8, 78.8,77.9% Fi: 1,2,3,4 yr:60.2, 56.2, 54.9, 52.3%	7
Nordmark 2006 [102]	Sweden	Cohort	*N* = 110 RA patients treated by a multidisciplinary team in addition to usual medical treatment	0.5	1995–98	n.a	18–60	75	Baseline: 66.4%; full-time 59.1%; part-time: 7.3% 24 months follow-up: 88.2%; full-time: 67.3%; part-time: 20.9%	6
Smolen 2006 [109]	The Netherlands	Cohort	*N* = 856 patients with early RA from an RCT-study of medical treatment follow-up 54 week	0.9	n.a	n.a	< 64 (47)	72	Baseline: 64% Full-time 53%; Part-time 11% Follow-up (54 w): 67.1%	6
Eberhardt 2007 [93]	Sweden	Cohort	*N* = 148 RA at baseline; from one department of rheumatology. After 15 yr *N* = 63	1	1985–89	80.9	48 (employed) 58 (unemployed)	64.2	Baseline 72.3% Follow-up after 15 yr:54%	5
Reisine 2007 [107]	US	Cohort	*n* = 48 and *n* = 91 female RA patients, employed at baseline diagnosed during the last yr selected from two bigger cohorts of RA patients	< 1.5	1987–98	n.a	> 18	100	Baseline: 100% Follow-up: 71.9%	5
Verstappen 2007 [112]	The Netherlands	Cohort	*N* = 148 in working age at start of the study; *N* = 63 (15 yr follow-up)	< 2 yr at study start	1985–2004	81	18–59 (48/54)	64	Study entry72.3% After 5 yr: 65% After 10 yr: 61% After 15 yr: 54%	6
Verstappen 2007 [112]	The Netherlands	Cohort	*N* = 461; 294 in working age	6.4	1999–2000	80	< 65	72	1 yr of follow-up: 33% 2 yr of follow-up: 26.2%	6
Azevedo 2008 [33]	Brazil	Cross sectional	*N* = 192 RA patients from one out-patient clinic in Sau Paulo	9.8	Feb-nov 2005	n.a	18–65 (47.4)	86	43.2%	7
Bejano 2008 [91]	UK	Cohort	*N* = 115 RA patients *n* = 61 Adalimumab + MTX *n* = 54 placebo + MTX	< 2	56-week follow-up	77.7	(47)	56.5	Baseline: 100% employed: Follow-up: Adalimumab + MTX: 77% Placebo + MTX: 46.3%	6
Han 2008 [121]	Austria, Canada, Denmark, France, Germany, Netherlands, Sweden, UK, US	RCT	*N* = 1222 patients < 65 yr from two double-blinded, randomized, controlled studies of patients with RA MTX: never or incomplete	81% > 3 yr	n.a	n.a	20–65	71.9–77.8	Baseline: Never MTX: 66.5% MTX: Incomplete responders: Early RA: 61.6% Long-standing RA: 47.1% 54-week evaluation: Never MTX: 59.5% MTX: Incomplete responders: Early RA: 54.8% Long-standing RA: 43.0%	6
Shanahan 2008 [71]	Australia	Cross sectional	*N* = 497 RA patients from one city	10.7	n.a	60.6	18–65	70	RA patients 51.1% General population:93%	7
Zhang 2008 [86]	Canada	Cross sectional	*N* = 389 RA patients treated with Adalimumab	12	n.a	44	(55)	78	36%	7
Zirkzee 2008 [117]	The Netherlands	Cohort	*N* = 69 from a cohort of 313 patients with early RA followed for 12 months	0.3	n.a	22	18–64	55	Study entry: 49% 12 months follow-up: 42%	6
Halpern 2009 [94]	US	Cohort	*N* = 1233 from a cohort receiving adalimumab or DMARD followed 24 months from Europe, Australia, and Canada	12–13	n.a	n.a	(54–57)	75.8	Baseline: 27.4% Follow-up: 12 months: Total:14.5% Adalimumab: 24.1% DMARD: 8.3% 24 months: Total:11.7% Adalimumab: 22.2% DMARD: 4.8%	6
Hoving 2009 [96]	The Netherlands	Cohort	*N* = 59 RA patients treated with Adalimumab	10.7	2004–6	n.a	(49)	76.3	Baseline: 44.1% 6 months follow-up: 35.6%	5
Osterhaus 2009 [67]	Austria, Czech Republic, US)	Cross sectional	*N* = 220 randomly selected to a 24-week multicenter RCT study of certolizumab pegol or placebo	9.5	2003–4	n.r	19–62	84	Baseline 38.6%	5
Hazes 2010 [53], Kavanaugh 2009 [56]	The Netherlands US	Cross sectional	*N* = 982 (RAPID 1); *N* = 619 (RAPID 2) multicenter, double-blind, placebo-controlled trial of certolizumab Pegol with MTX on work productivity	6	n.a	n.a	(52)	82–83	Baseline: 38.4% RAPID 1: Total 41.6% RAPID 2: Total 39.8%	5
Herenius 2010 [95]	The Netherlands	Cohort	*N* = 126 RA patients	6.4	n.a	n.a	18–62 (49)	73.8	50%	6
Sokka 2010 [18]	Finland	Cross sectional	5493 RA patients < 65 yr from a multinational study from 86 sites in 36 countries with self-reported employment status	11	2005–9	n.a	< 65 yr	80	Before RA: Males: 85% (57–100%) Females: 64% (19–87%) After RA:47.2%	7
Van Vollenhoven 2010 [110]	Sweden	Cohort	*N* = 664 (baseline) RA patients in a RCT study of MTX (*n* = 214); Adalimumab + MTX (*n* = 219); Adalimumab (*n* = 231); 2-yr multicenter study	0.8	n.a	83	(52)	75	Baseline: Total: 55%	5
Verstappen 2010 [119]	The Netherlands	Cohort	*N* = 3291 RA patients treated with anti TNF and 379 RA controls: 3 yr follow-up	Anti-TNF:12 Controls 8	n.a	n.a	(50–52)	76–77	Anti-TNF: baseline: 37.6%; follow-up 34.2% Controls: baseline; 46.7%; follow-up 44.3%	5
Bodur 2011 [37]	Turkey	Cross sectional	*N* = 49 RA from an outpatient clinic in Ankara	9.7	Within 6 months	n.a	46.6	63.3	22.4%	4
Nikiphorou 2012 [101]	UK	Cohort	*N* = 877 (baseline) from nine outpatients’ clinics; median 10 yr follow-up (*N* = 591)	< 2	1986–98	67.4	< 60	68	Baseline: 67% Follow-up: 42.6%	5
Da Rocha Castelar Pinheiro 2013 [88]	Brazil	Cross sectional	*N* = 526 RA patients	6.5	2007	n.a	51	80	29%	4
Smolen 2012 [72]	The Netherlands	Cross sectional	*N* = 520 with available employment data from a cohort with early progressive RA (RCT-study of medical treatments in *n* = 638)	0.7	n.a	81.5	(52)	73.8	56.9%	6
Mattila 2014 [61]	Eleven countries in EU	Cross sectional	*N* = 1061; 100 RA patients from each of the countries answered a telephone interview	> 2	n.a	n.a	(49–57)	74–82	30% (Finland) 57% (Italy)	5
McWilliams 2014 [99]	UK	Cohort	*N* = 1235 from The Early RA Network, ERAN inception cohort study from 22 centers in the UK and Ireland	0–10	2002–12	83.8	47–98 (58)	68	Baseline 47% Follow-up: 10% had lost job 37% employed	6
Tamborenea 2015 [74]	Argentina	Cross sectional	*N* = 450 consecutive RA patients from 31 urban rheumatology clinics from 11 provinces	> 0.5	n.a	n.a	(48–49)	82.6	45.5%	5
Bertin 2016 [36]	France	Cross sectional	*N* = 488 recruited from 90 rheumatologists in hospital or office practice	12.2	2012–13	n.a	< 60	84.4	74.6%	7
Pieringer 2016 [68]	Austria	Cross sectional	*N* = 3847 RA patients from 15 countries from four continents (COMORA-study)	9.4	2011–12	n.r	(57)	81	31.4%	5
Wan 2016 [81]	Singapore	Cross sectional	*N* = 108 RA from one rheumatology clinic	7.6	2013–14	87.1	56.4	79.6	Full-time: 29.7% Part.time:14.8%	6
Lapcevic 2017 [60]	Serbia	Cross sectional	*N* = 409 RA patients, multicenter study in 22 health institutions	12	2014	82.8	(58)	87	20.1%	5
Rosa-Gocalves 2018 [69]	Portugal	Cross sectional	*N* = 154 RA consecutive patients from one hospital	16	2013–14	n.a	(56)	87.7	33.8%	5
Van der Zee-Neuen 2017 [76]	The Netherlands	Cross sectional	*N* = 2395 RA patients < 60 yr from 17 countries from five continents (COMORA-study)	n.a	n.a	n.a	18–60 (48)	84	45% (18.2–70.6%)	7
Vazquez-Villegas 2017 [111]	Mexico	Cohort	*N* = 614 RA patients	7	1992–2012	n.a	> 18 (42)	83	60.6%	6
Anno 2018 [32]	Japan	Cross sectional	*N* = 191 RA patients and 191 sex matched without RA (control group) from one university hospital	18.1	2010	n.a	> 20	84.5	RA: Full-time 18.3%; part-time 16.2% Controls: Full-time 17.8%; part-time 21.5%	8
Berner 2018 [35]	Austria	Cross sectional	100 seropositive RA from one outpatient clinic	6.5	2015–16	71.4	18–65 (53)	66	59%	8
Chen 2018 [41]	Taiwan	Cross sectional	*N* = 330 RA from 50 rheumatologists in Taiwan	13.2	n.a	n.a	60	74	19.1% among persons in working age	5
Gomes 2018 [51]	Brazil	Cross sectional	*N* = 133 with RA from a municipality in south Brazil	n.a	2014–15	55.1	20–59	82.7	48.8%	8
Fara 2019 [49]	Argentina	Cross-sectional	*N* = 126 with RA, applicants for disability certificate	10	2012–16	n.a	> = 16 (55)	79	At application time 36%	6
Xavier 2019 [82]	Argentina Brazil Colombia Mexico	Cross sectional	*N* = 290 from 18 rheumatology public and private clinics from Argentina *N* = 75; Brazil *N* = 68; Colombia *N* = 72; Mexico *N* = 75	n.a	2012–15	n.a	21–50 (43.7)	90	Argentina 72.6% Brazil 44.2% Colombia 62.5% Mexico 57.3%	5
Gwinnutt 2020 [122]	UK	Cohort	*N* = 463 MTX-starters; *N* = 260 biologic starters	0.5;5	2008–12	n.a	18–65	68;77	Baseline: 100% 1 yr follow-up: 89%	4
Intriago 2020 [55]	Ecuador	Cross sectional	*N* = 395 RA from one clinic	13.8	2019	n.a	51.4	87.8	40.5%	5
Sacilotto 2020 [70]	Brazil	Cross sectional	*N* = 1115 from a prospective cohort study of RA patients from 11 public health care centers	12.7	n.a	n.a	> 18 (56.7)	90	26.8%	5
Syngle 2020 [73]	India	Cross sectional	*N* = 52 RA patients from an outpatient department	7	2017–18	n.a	29–60 (46)	77	53.8%	6
Zolnierczyk-Zreda 2020 [87]	Poland	Cross sectional	*N* = 282 RA outpatients from 3 hospitals	n.a	n.a	100	50.6	80	63.5	8
Al-Jabi 2021 [29]	Palestine	Cross sectional	*N* = 300 from all rheumatology clinics in a part of Palestine	6	2012	n.a	(49)	76.3	26.3%	5
Morf 2021 [65]	Germany Brazil	Cross sectional	*N* = 176 RA from Germany *N* = 91 RA from Brazil from two outpatient clinics	Germany 14.4 Brazil 15.9	2011–12	n.a	Germany 62.4 Brazil 56.3	Germany 78.4 Brazil 92.3	Germany 31.8% Brazil 35.2%	5
Tanaka 2021 [75]	Japan	Cross sectional	*N* = 357 from 82 centers	6.9	2013–17	n.a	58	82.1	Full-time 24.6; Part-time 14.3 Responders: Full-time 28.3; Part-time 19.1 Non-responders: Full-time 20.3; Part-time 11.9	6
Yates 2021 [84]	UK	Cross sectional	*N* = 7455 RA from 209 secondary rheumatology care units	Newly diagnosed	2018–19	n.a	56.7	62.3	48%	4
Dejaco 2022 [47]	Austria	Cross sectional	*N* = 95 RA baseline; 59 at follow-up multicentre study	n.a	n.a	n.a	54.8	82.1	48.4%	5
Hamdeh 2022 [52]	Palestine	Cross sectional	*N* = 285, a sample of 1042 RA patients from rheumatology clinics	9.1	2012	n.a	8529	81.1	23.5%	5
Hulander 2022 [54]	Sweden	Cross sectional	*N* = 50 RA from a RCT study from one outpatient clinic	18.3	2017	84	62.3	81	64%	5
Lahiri 2022 [59]	Singapore	Cross sectional	*N* = 121 RA from a single center RDCT study	5.5	2016	86	56.6	86.3	46.3%	5
Li 2022 [80]	China	Cross sectional	*N* = 215 RA from a single out- and inpatient Rheumatology department	10.3	2017–20	69.4	55.4	90.2	Employed 33% Unemployed30% Retired 37%	6
McQuillan 2022 [62]	US	Cross sectional	*N* = 854 from National Rheumatoid Arthritis Study	10.2	1988–98	n.a	58	78	41%	5
Yajima 2022 [83]	Japan	Cross sectional	*N* = 165 RA from 4 outpatient clinics	4.5	2013–14	91.2	64	86.1	23%	6

*n.a.* not analysed, *n.r.* not relevant, *RCT* randomized controlled clinical trial, *yr* year

### General description of study participants

On average, patients with RA were 51 years old, with an age range spanning from 42 to 64 years. Furthermore, the female population accounted for 75.9% of the patient cohort, with a range from 41 to 92%. The duration of the disease at study entry exhibited significant variability, ranging from less than one year up to more than 18 years on average.

### Employment rate

At disease onset, the employment rate was 78.8% (weighted mean, range 45.4–100), at study entry 47.0% (range 18.5–100), and during the follow-up period 40.0% (range 4–88.2), as shown in Table 2. Notably, a comparative analysis of the employment rates between Europe and North America indicated no substantial difference (*p* = 0.93). However, the comparison between Europe, North America and ‘other continents’ did yield significant differences (or nearly differences) with *p*-values of 0.003 and 0.08, respectively.

**Table 2:** Employment rate in patients with RA, by continent (weighted mean, SD, range)

Region	Weighted mean (%)	SD	Range
Disease onset employment			
Europe	60.3	24.9	45.4–100
North America	83.3	10.0	65–100
Other			
Total	78.8	16.2	45.4–100
Study entry employment			
Europe	43.5	15.9	18.5–89.0
North America	54.2	22.8	27.4–100
Other	41.2	12.2	20.5–60.6
Total	47.8	18.3	18.5–100
Follow-up employment			
Europe	42.7	13.9	29.3–88.2
North America	33.6	26.1	4–71.9
Other			
Total	40.0	19.7	4–88.2

The employment rate exhibited no change, when comparing studies from the 1980s through to 2022. Specifically, the weighted mean for the years 1981–2000 was 49.2%, aligning closely with the corresponding figures for the years 2001–2010 (49.2%) and 2011–2022 43.6%. These findings were statistically non-significant, with *p*-values of 0.80 for comparison between year 1981–2000 and 2001–2010; 0.66 for 2001–2010 and 2011–2022, and 0.94 for 1981–2000 and 2011–2022, shown in Figure S1, see Additional file.

Among the studies included in the analysis, nineteen studies included data of employment at follow-up, with durations ranging from 1 to 20 years, Table 2. For instance, Jäntti, 1999 [97] reported an employment rate 69% one year after disease onset, which gradually declined to 50% after 15 years and further to 20% after 20 years. Similarly, Mäkisara, 1982 [63] demonstrated that 60% of the patients were employed 5 years after disease onset, 50% after 10 years, and 33% after 15 years. Nikiphorou, 2012 [101] reported an employment rate of 67% at study entry, which decreased to 43% after 10 years.

In addition, seven studies included data of employment rate among patients comparing different medical treatments [18, 44, 56, 91, 105, 110, 119]. These studies indicated that, on average, 55.0% (weighted mean) of the patients were employed after receiving treatment with MTX, while 42.8% after undergoing treatment with a combination of MTX + Adalimumab (all patients were employed at disease onset in these specific studies).

### Predictors for employment

Information of normative comparison data to use for meta-analysis of predictors for employment at study entry was available for age, gender, educational level, race, job type, comorbidities, MTX at any time, biological treatment at any time, prednisolone at any time, disease duration, HAQ score, joint pain (VAS-score), and disease activity (DAS28 score). Predictors for employment at study entry was being younger /age below 50 years, being a male, higher educational level (college or more), non-manual work, having no comorbidities, no medical treatment, short disease duration, and low HAQ score, VAS-score, or DAS28 score. Heterogeneity was small for age, gender, medical treatment, and moderate for educational level, and job type as indicted by the I^2^ values, Table 3, and shown in detail in Figures S2, S3, S4, S5, S6, S7, S8, S9, S10, S11, S12, S13, S14, S15 and S16, see Additional file.

**Table 3:** Predictors for employment among patients with RA

**Predictor**	Studies	Participants	Chi^2^	*p*	I^2^ (%)	OR	95% CI
Age (< = 50/ > 50 years)	5	2623	74.4	< 0.001	95	3.56	2.97, 4.26
Gender (female/male)	28	16248	74.1	< 0.001	64	0.58	0.53, 0.62
Educational level (college/below college)	14	5117	20.2	0.09	35	2.25	1.96, 2.59
Race (Caucasian/not Caucasian)	8	3197	12.0	0.10	42	1.13	0.90, 1.42
Job type (not manual/manual)	8	2366	13.8	0.06	49	2.38	1.89, 3.00
Comorbidities (not present/present)	4	1932	4.2	0.25	28	1.74	1.41, 2.14
MTX ever (no/yes)	5	3191	5.44	0.25	26	0.87	0.73, 1.03
Biological treatment ever (no/yes)	6	11960	132	< 0.001	96	0.89	0.81, 0.98
Prednisolone ever (no/yes)	2	633	1.05	0.3	5	0.41	0.25, 0.68
**Predictor**	Studies	Participants	Mean difference				95% CI
Age (low/high age)	17	4361	-6.59				-7.03, -6.15
Disease duration (short/long)	8	1998	-1.54				-2.05, -1.03
HAQ score (low/high)	5	1652	-0.48				-0.55, -0.40
Joint pain, VAS score (low/high)	4	906	-0.92				-1.25, -0.59
SF 36 (low/high)	2	309	7.57				5.03, 10.1
DAS 28 (low/high)	2	568	-0.27				-0.48, -0.06

### Assessment of quality of included studies

All studies were subject to rigorous quality assessment. These assessments resulted in categorisation of either medium quality (*n* = 64; 70%) or high-quality studies (*n* = 27; 30%), with no studies falling into the low-quality category. The quality assessment is shown in Tables 4 and 5.

**Table 4:**
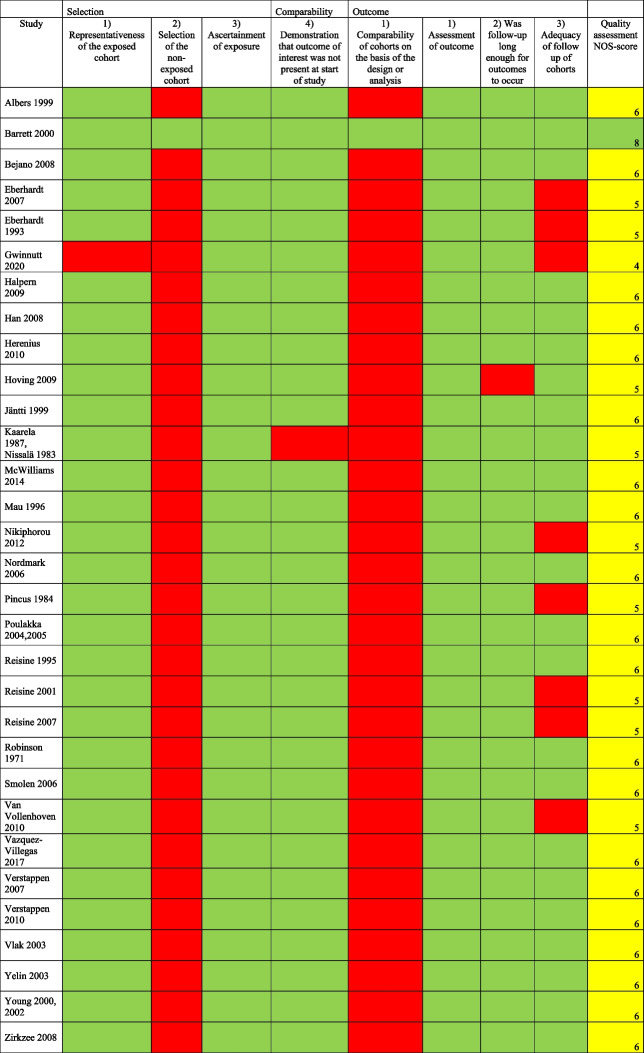
Assessment of quality of the included cohort studies. NOS heat map [89–103, 106–117, 119, 121, 122, 130]

**Table 5:**
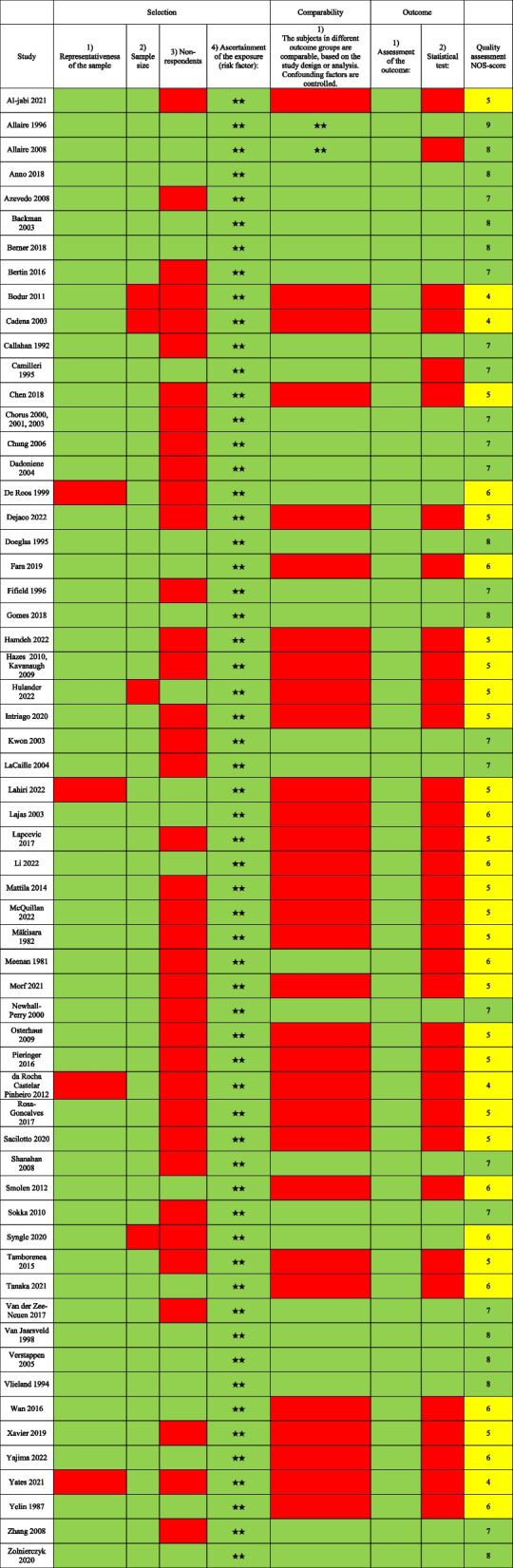
Assessment of quality of the included cross-sectional studies. NOS heat map [13, 18, 29–33, 35–60, 62–68, 70–87, 129]

Notably, many studies were characterised by several common attributes, including cross-sectional study design, single-centre-settings, relatively small sample sizes, and the reliance on self-reported patient data. When including only the high-quality studies in the analyses, the employment rates at study entry changed from 47% (weighted mean, all studies) to 50% (weighted mean, high quality studies).

## Discussion

### Key findings

This systematic review has identified a decline in the employment rate among patients with RA, with a notable decrease from disease onset during the study entry to follow-up, where only half of the patients were employed. These findings corroborate earlier research that indicated a substantial decline in employment rates among patients with RA over time. Notably, previous studies have reported that approximately one third of patients with RA stopped working within 2 to 3 years after disease onset, and more than half was unable to work after 10 to 15 years [23, 63, 93, 97, 101]. Only few studies have included data from the general population, comparing the employment rates with the rates for patients with RA [89, 90]. Comparisons with the general population further underscored the challenges faced by RA patients, as their employment rates were consistently lower.

Despite changes in medical treatment, social security systems, and societal norms over the past decades, there was no significant improvement in the employment for patients with RA. This pattern aligns with data from the Global Burden of Disease studies, highlighting the persistent need for novel approaches and dedicated efforts to support patients with RA in sustaining employment [2, 123]. Recent recommendations from EULAR (European Alliance of Associations for Rheumatology) and ACR (American College of Rheumatology) have emphasized the importance of enabling individuals with rheumatic and musculoskeletal diseases to engage in healthy and sustainable work [17, 124, 125].

While different countries possess different social laws and health care systems for supporting patients with chronic diseases, the variations in the weighted mean of employment rates across countries were relatively minor.

In the meta-analysis, one of the strongest predictors for maintaining employment was younger age at disease onset [43, 51, 101, 116]. Verstappen, 2004 found that older patients with RA had an increased risk of becoming work disabled, potentially caused by the cumulative effects of long-standing RA, joint damage, and diminished coping mechanisms, compared to younger patients [23].

More women than men develop RA, however this study showed that a higher proportion of men managed to remain employed compared to women [18, 36, 42, 43, 46, 62, 71, 89, 101, 116]. Previous studies have shown inconsistent results in this regard. Eberhart, 2007 found that a significantly higher number of men with RA worked even though there was no difference in any disease state between the sexes [93]. De Roos,1999 showed that work-disabled women were less likely to be well-educated and more likely to be in a nonprofessional occupation than working women. Interestingly, there was no association of these variables among men. Type of work and disease activity may influence work capacity more in women than in men [46]. Sokka, 2010 demonstrated a lower DAS28 and HAQ-score in men compared to women among the still working patients with RA, which indicated that women continued working at higher disability and disease activity levels compared with men [18].

Disease duration also played a significant role as a predictor of employment outcomes [33, 36, 45, 71, 77, 86, 102, 111]. Longer disease duration correlate with decreased employment likelihood, which could be attributed to older age and increased joint damage and disability in patients with longer-standing RA.

Higher educational levels were associated with a greater possibility of employment [30, 43, 45, 46, 51, 62, 86]. This is probably due to enhanced job opportunities, flexibility, lower physical workload, better insurance coverage, and improved health care for well-educated individuals. This is further supported by the fact that having a manual work was a predictor for not being employed [30, 39, 43–45].

Furthermore, health-related quality of life, as measured by SF 36, lower disease activity (DAS28 scores), reduced joint pain (VAS-score), and lower disability (HAQ score) were additionally predictors for being employed [33, 35, 36, 45, 71, 86]. This support the statement that the fewer symptoms from RA, the greater the possibility of being able to work.

The results showed that the presence of comorbidity was a predictor for not being employed, aligning with findings from previous studies that chronic diseases such as cardiovascular disease, lung disease, diabetes, cancer, and depression reduced the chances of being employed [126]. Moreover, the risk of exiting paid work increased with multimorbidity [127].

While limited data were available for assessing the impact of treatment on employment, indications suggested that patients with RA were receiving medical treatments, such as MTX or biological medicine, were more likely to be unemployed. One possible explanation for this phenomenon could be that patients with RA, who were receiving medical treatment, had a more severe and a longer duration of RA compared to those, who had never been on medical treatment. However, the scarcity of relevant studies necessitates caution when drawing definitive conclusions in this regard.

Therefore, the predictors for employment found in this review were being younger, being a male, having higher education, low disease activity, low disease duration, and being without comorbidities. This is supported by previous studies [93, 116]

In summary, this review underscores the importance of managing disease activity, offering early support to patients upon diagnosis, and reducing physically demanding work to maintain employment among patients with RA. Achieving success in this endeavour requires close cooperation among healthcare professionals, rehabilitation institutions, companies, and employers. Furthermore, it is important that these efforts are underpinned by robust social policies that ensure favourable working conditions and provide financial support for individuals with physical disabilities, enabling them to remain active in the labour market.

### Strengths and limitations

The strength of this review and meta-analysis lies in the inclusion of a large number of articles originating from various countries. Furthermore, the data showed a consistent employment rate in high quality studies compared to all studies. However, there are some limitations to this review. No librarian was used to define search terms and only three databases were searched. Furthermore, the initial search, selection of articles, data extraction, and analysis was undertaken only by one author, potentially leading to the omission of relevant literature and data. The review also extended back to 1966, with some articles from the 1970s and 1980s included. Given the significant changes in medical treatment, social security systems, and society over the past decades, the generalizability of the findings may be limited.

Moreover, the majority of studies did not include a control group from the general population, which limited the ability to compare employment rates with the general population in the respective countries. Many studies were cross-sectional in design, which limits the evidence of causality between employment rate and having RA. However, the employment rate was approximately the same in high quality studies compared to all studies, which supports an association. A substantial number of studies relied on self-reported employment rates, introducing the potential for recall bias. Additionally, many studies did not account for all relevant risk factors for unemployment failing to control for all relevant confounders.

EULAR have made recommendation for point to consider when designing, analysing, and reporting of studies with work participation as an outcome domain in patients with inflammatory arthritis. These recommendations include study design, study duration, and the choice of work participation outcome domains (e.g., job type, social security system) and measurement instruments, the power to detect meaningful effects, interdependence among different work participation outcome domains (e.g., between absenteeism and presentism), the populations included in the analysis of each work participation outcome domain and relevant characteristics should be described. In longitudinal studies work-status should be regularly assessed and changes reported, and both aggregated results and proportions of predefined meaningful categories should be considered [128]. Only some of the studies in this review met the requirements for high quality studies. In both older and newer studies methodological deficiencies persisted in study design, analysis, and reporting of results, as recommended by EULAR.

## Perspectives for future studies

Future research in this area should focus on developing and evaluating new strategies to address the ongoing challenges faced by patients with RA in maintaining employment. Despite many initiatives over the years, there has been no success in increasing employment rates for patients with RA in many countries. Therefore, there is a pressing need for controlled studies that investigated the effectiveness of interventions such as education, social support, and workplace adaptations in improving employment outcomes for these individuals.

## Conclusion

This systematic review underscores the low employment rate among patients with RA. Key predictors of sustained employment include being younger, having higher educational level, short disease duration, and lower disease activity, along with fewer comorbidities. Importantly, the review reveals that the employment rate has not changed significantly across different time periods. To support patients with RA in maintaining their employment, a comprehensive approach that combines early clinical treatment with social support is crucial. This approach can play a pivotal role in helping patients with RA stay connected to the labour market.

### Supplementary Information

**Additional file 1: Figure S1.** Employment; year of investigation.

**Additional file 2: Figure S2.** Forest Plot of Comparison: Predictors for employment. Outcome: Younger or older age.

**Additional file 3: Figure S3.** Forest Plot of Comparison: Predictors for employment. Outcome: >50 yr or <50 yr of age.

**Additional file 4: Figure S4.** Forest Plot of Comparison: Predictors for employment. Outcome: Gender: Male or Female.

**Additional file 5: Figure S5.** Forest Plot of Comparison: Predictors for employment. Outcome: Educational level: no college education or college education or higher.

**Additional file 6: Figure S6.** Forest Plot of Comparison: Predictors for employment. Outcome: no comorbidities present or one or more comorbidities present.

**Additional file 7: Figure S7.** Forest Plot of Comparison: Predictors for employment. Outcome: Ethnicity: Caucasian or other than Caucasian.

**Additional file 8: Figure S8.** Forest Plot of Comparison: Predictors for employment. Outcome: Short or long disease duration.

**Additional file 9: Figure S9.** Forest Plot of Comparison: Predictors for employment. Outcome: Low or high Health Assessment Questionnaire, HAQ-score.

**Additional file 10: Figure S10. **Forest Plot of Comparison: Predictors for employment. Outcome: Low or high VAS-score.

**Additional file 11: Figure S11.** Forest Plot of Comparison: Predictors for employment. Outcome: Job type: blue collar workers or other job types.

**Additional file 12: Figure S12.** Forest Plot of Comparison: Predictors for employment. Outcome: No MTX or MTX.

**Additional file 13: Figure S13.** Forest Plot of Comparison: Predictors for employment. Outcome: No biological or biological.

**Additional file 14: Figure S14.** Forest Plot of Comparison: Predictors for employment. Outcome: No prednisolone or prednisolone.

**Additional file 15: Figure S15.** Forest Plot of Comparison: Predictors for employment. Outcome: Low or high DAS score.

**Additional file 16: Figure S16.** Forest Plot of Comparison: Predictors for employment. Outcome: Low or high SF 36-score.

## Data Availability

The datasets used and/or analyzed during the current study are available in the supplementary file.

## Abbreviations

RA: Rheumatoid arthritis
MTX: Methotrexate
Yr: Year
NOS: Newcastle Ottawa Quality Assessment Scale
SD: Standard deviation
n.a.: Not analyzed
n.r.: Not relevant
yr: Year
DAS: Disease activity
HAQ: Health Assessment Questionnaire
VAS: Visual analog scale for pain
EULAR: European Alliance of Associations for Rheumatology
ACR: American College of Rheumatology
