# Supplementary figures and images for: Employment of patients with rheumatoid arthritis - a systematic review and meta-analysis
Source: BMC Rheumatol. 2023 Nov 14;7:41. doi: 10.1186/s41927-023-00365-4 (PMC10644429; doi:10.1186/s41927-023-00365-4)

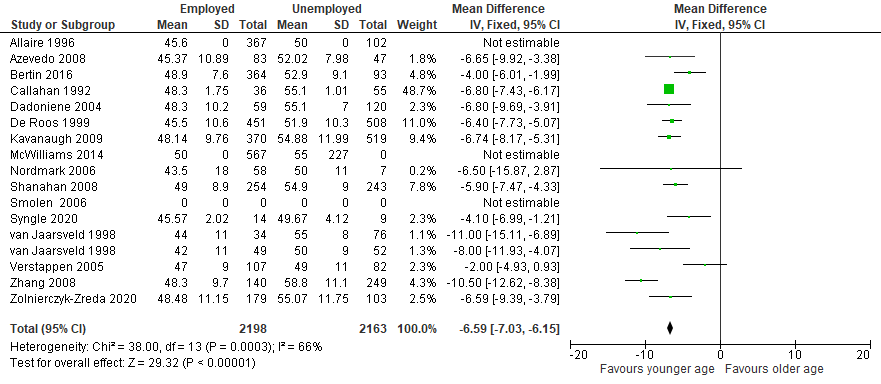

Supplement: Supplementary file 2 — Additional file 2: Figure S2. Forest Plot of Comparison: Predictors for employment. Outcome: Younger or older age. [file 41927_2023_365_MOESM2_ESM.docx]

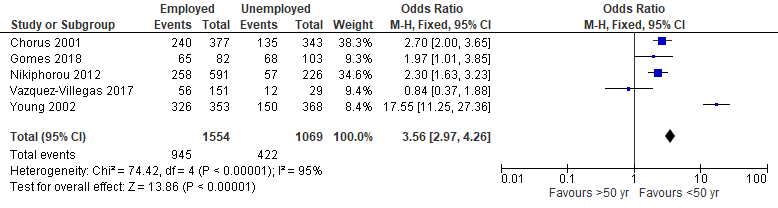

Supplement: Supplementary file 3 — Additional file 3: Figure S3. Forest Plot of Comparison: Predictors for employment. Outcome: >50 yr or <50 yr of age. [file 41927_2023_365_MOESM3_ESM.docx]

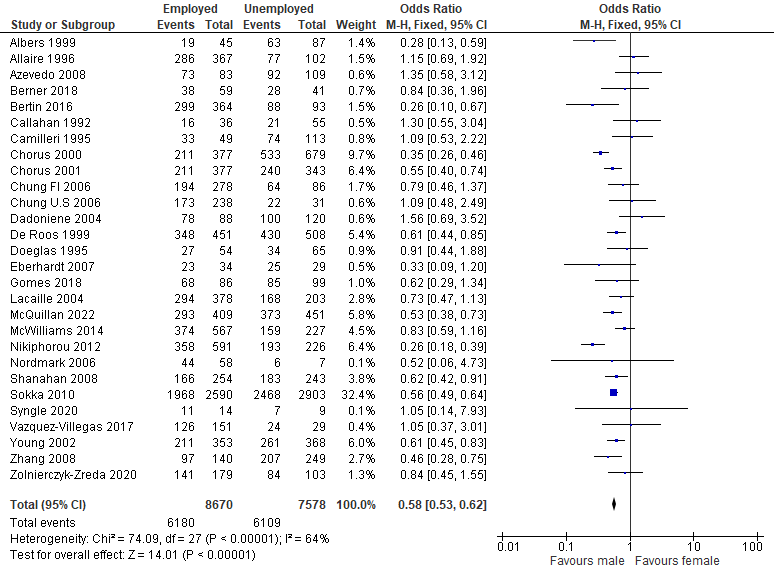

Supplement: Supplementary file 4 — Additional file 4: Figure S4. Forest Plot of Comparison: Predictors for employment. Outcome: Gender: Male or Female. [file 41927_2023_365_MOESM4_ESM.docx]

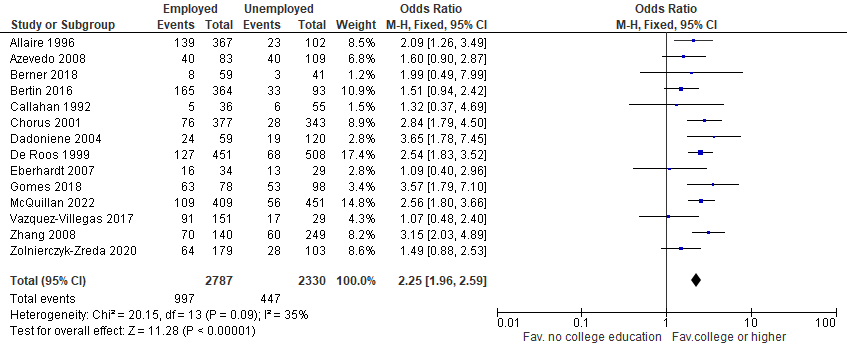

Supplement: Supplementary file 5 — Additional file 5: Figure S5. Forest Plot of Comparison: Predictors for employment. Outcome: Educational level: no college education or college education or higher. [file 41927_2023_365_MOESM5_ESM.docx]

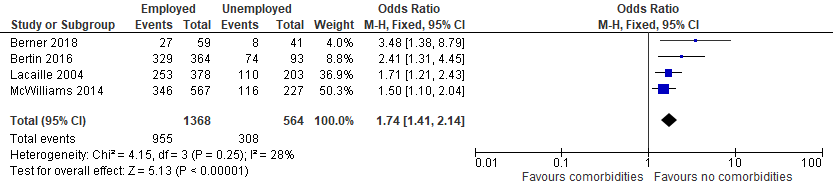

Supplement: Supplementary file 6 — Additional file 6: Figure S6. Forest Plot of Comparison: Predictors for employment. Outcome: no comorbidities present or one or more comorbidities present. [file 41927_2023_365_MOESM6_ESM.docx]

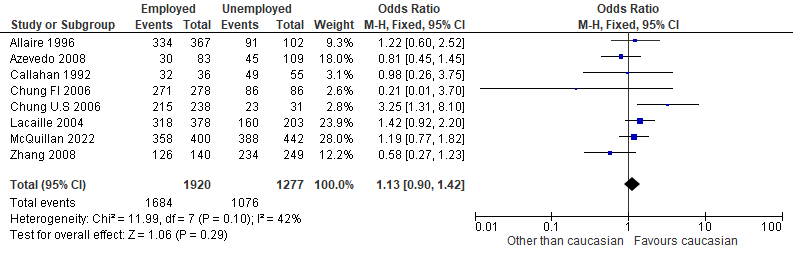

Supplement: Supplementary file 7 — Additional file 7: Figure S7. Forest Plot of Comparison: Predictors for employment. Outcome: Ethnicity: Caucasian or other than Caucasian. [file 41927_2023_365_MOESM7_ESM.docx]

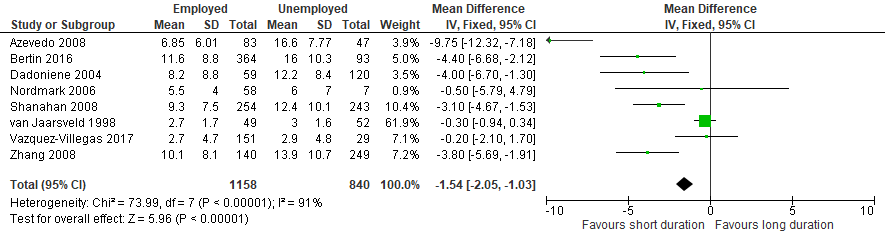

Supplement: Supplementary file 8 — Additional file 8: Figure S8. Forest Plot of Comparison: Predictors for employment. Outcome: Short or long disease duration. [file 41927_2023_365_MOESM8_ESM.docx]

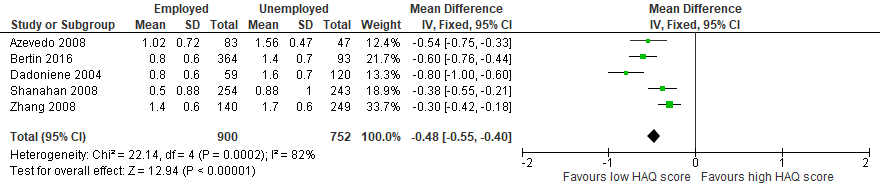

Supplement: Supplementary file 9 — Additional file 9: Figure S9. Forest Plot of Comparison: Predictors for employment. Outcome: Low or high Health Assessment Questionnaire, HAQ-score. [file 41927_2023_365_MOESM9_ESM.docx]

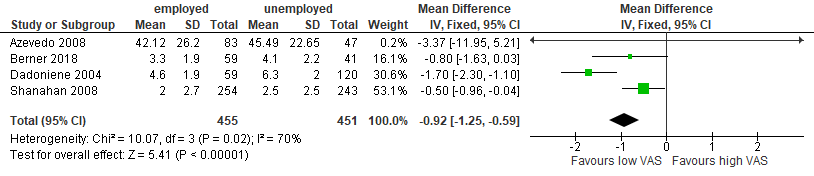

Supplement: Supplementary file 10 — Additional file 10: Figure S10. Forest Plot of Comparison: Predictors for employment. Outcome: Low or high VAS-score. [file 41927_2023_365_MOESM10_ESM.docx]

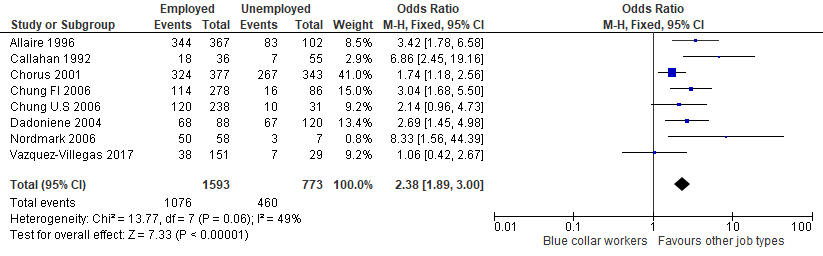

Supplement: Supplementary file 11 — Additional file 11: Figure S11. Forest Plot of Comparison: Predictors for employment. Outcome: Job type: blue collar workers or other job types. [file 41927_2023_365_MOESM11_ESM.docx]

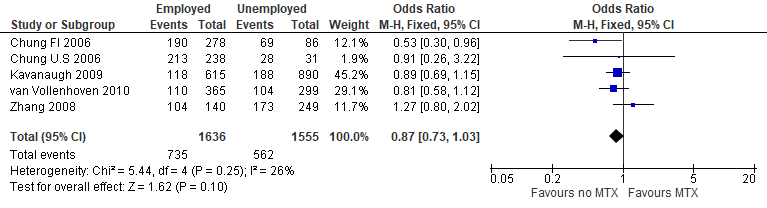

Supplement: Supplementary file 12 — Additional file 12: Figure S12. Forest Plot of Comparison: Predictors for employment. Outcome: No MTX or MTX. [file 41927_2023_365_MOESM12_ESM.docx]

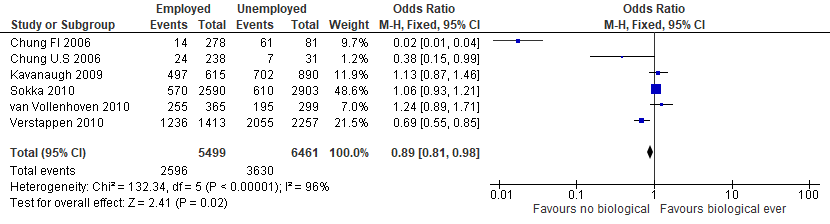

Supplement: Supplementary file 13 — Additional file 13: Figure S13. Forest Plot of Comparison: Predictors for employment. Outcome: No biological or biological. [file 41927_2023_365_MOESM13_ESM.docx]

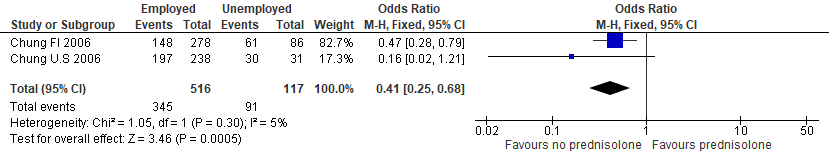

Supplement: Supplementary file 14 — Additional file 14: Figure S14. Forest Plot of Comparison: Predictors for employment. Outcome: No prednisolone or prednisolone. [file 41927_2023_365_MOESM14_ESM.docx]

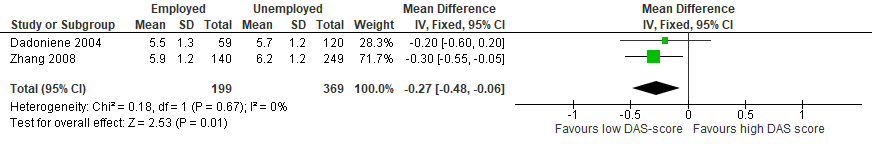

Supplement: Supplementary file 15 — Additional file 15: Figure S15. Forest Plot of Comparison: Predictors for employment. Outcome: Low or high DAS score. [file 41927_2023_365_MOESM15_ESM.docx]

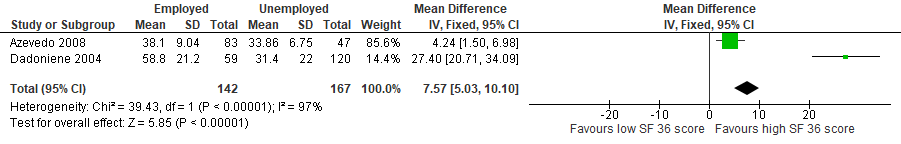

Supplement: Supplementary file 16 — Additional file 16: Figure S16. Forest Plot of Comparison: Predictors for employment. Outcome: Low or high SF 36-score. [file 41927_2023_365_MOESM16_ESM.docx]
