# Supplementary material for: Employment of patients with rheumatoid arthritis - a systematic review and meta-analysis
Source: BMC Rheumatol. 2023 Nov 14;7:41. doi: 10.1186/s41927-023-00365-4 (PMC10644429; doi:10.1186/s41927-023-00365-4)
Supplement: Supplementary file 2 [file 41927_2023_365_MOESM1_ESM.docx]

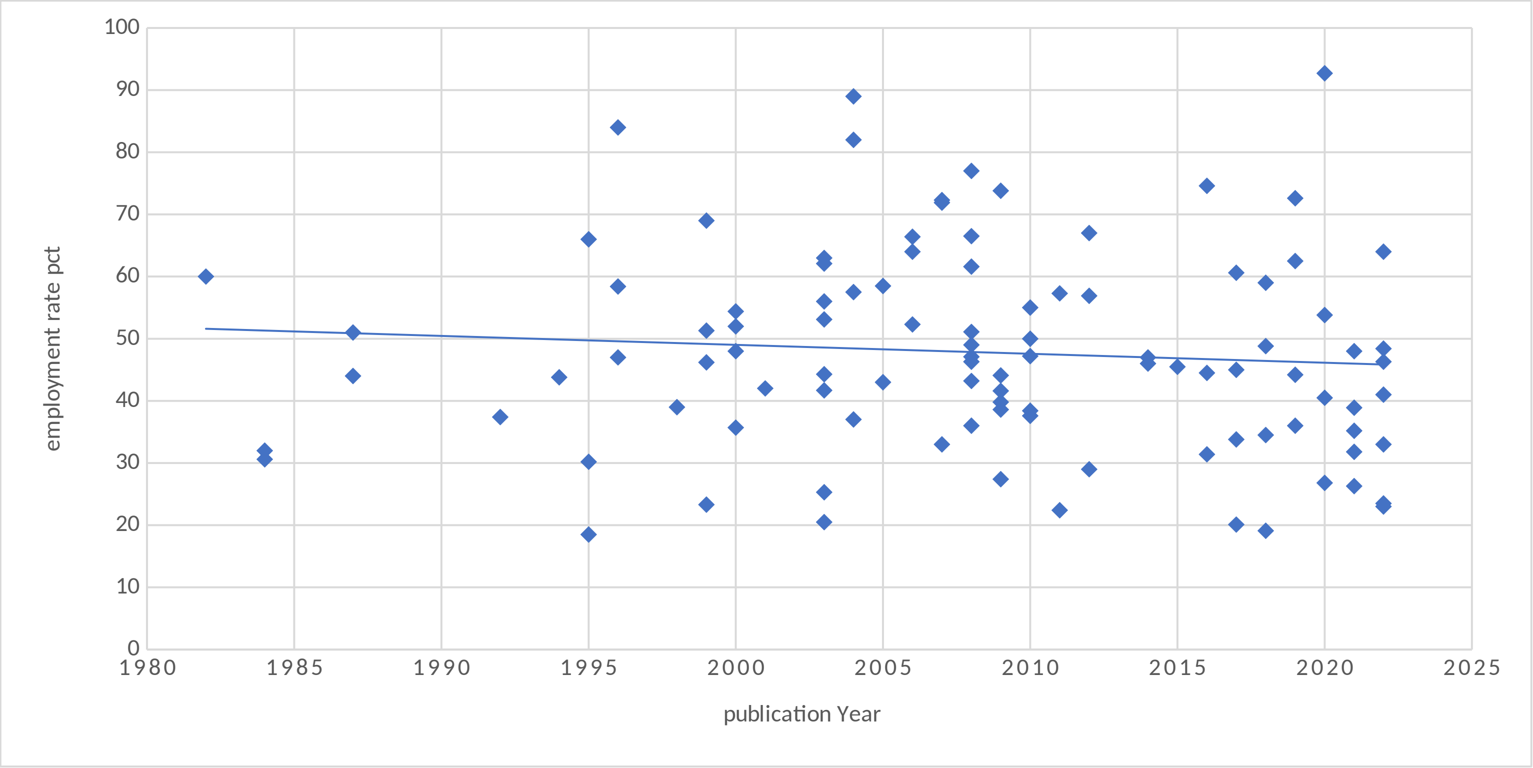
100
90
80
70
60
50
40
30
20
10
0
employment rate pct
1980
1985
1990
1995
2000
2005
2010
2015
2020
2025
publication Year
